# Four complete mitochondrial genomes of *Saurogobio* fishes (Cypriniformes: Gobionidae)

**DOI:** 10.1080/23802359.2019.1623126

**Published:** 2019-07-10

**Authors:** Jie Tong, Cuizhang Fu

**Affiliations:** Ministry of Education Key Laboratory for Biodiversity Science and Ecological Engineering, Coastal Ecosystems Research Station of the Yangtze River Estuary, Institute of Biodiversity Science and Institute of Eco-Chongming, School of Life Sciences, Fudan University, Shanghai, China

**Keywords:** *Saurogobio*, Cypriniformes, Gobionidae, phylogeny, East Asia

## Abstract

In this study, we determined complete mitochondrial genomes of *Saurogobio gracilicaudatus*, *S. xiangjiangensis*, *S. gymnocheilus*, and *S. lissilabris* so that these data could contribute to reconstruct interspecific phylogenetic relationships within the genus *Saurogobio*. The four mitochondrial genomes showed A + T bias (55.2–57.0%) of base compositions with the length from 16,594 to 16,608 bp. Phylogenetic relationships among *Saurogobio* fishes and their close relatives showed that the genus *Saurogobio* was a monophyletic group and it could be divided into two major groups.

*Saurogobio* fishes (Cypriniformes: Gobionidae *sensu* Tan and Armbruster 2018) are endemic to East Asia, including eight species (Tang et al. 2018). In this study, we determined complete mitochondrial genomes (mitogenomes) of *Saurogobio gracilicaudatus*, *S. xiangjiangensis*, *S. gymnocheilus*, and *S. lissilabris* so that these data could contribute to reconstruct interspecific phylogenetic relationships within the genus *Saurogobio*.

Sampled specimens were deposited in the Zoological Museum of Fudan University (FDZM), collected from three localities in China, including Huaihua City (27.55°N, 109.96°E) for *S. gracilicaudatus* (Voucher, FDZM-SGHUH20160915) and *S. xiangjiangensis* (FDZM-SXHUH20160915), Hengdong City (26.91°N, 113.18°E) for *S. gymnocheilus* (FDZM-SGYHD20180726), and Fengxin City (28.71°N, 115.38°E) for *S. lissilabris* (FDZM-SLFX20180727). Total genomic DNA were extracted from muscle tissues using a high-salt method (Miller et al. 1988). The Sanger sequencing was used to obtain all sequences, and each mitogenome was assembled by referring to the *S. dabryi* mitogenome (GenBank number: KU314696).

Four new mitogenomes (GenBank numbers: MK860909- MK860912) showed A + T bias (55.2–57.0%) of base compositions with the length from 16,594 to 16,608 bp, and displayed the same gene arrangements including 13 protein-coding genes, 2 rRNA genes, 22 tRNA genes, and 1 control region. The protein-coding genes utilized two types of start codons (ATG and GTG) and three types of stop codons (TAA, TAG, and T–). Eight pairs of adjacent genes displayed gene overlaps with variable size from 1 to 7 bp, and 13 pairs of adjacent genes showed gene intervals with variable size from 1 to 33 bp. Similar patterns in the gene arrangements, codon use, gene overlap, and gene interval have also been observed in published mitochondrial genomes of *Saurogobio* fishes (Wan et al. 2015; Li et al. 2018).

A phylogeny of *Saurogobio* fishes was reconstructed using 22 mitogenomes under six partitions, i.e. each codon of all protein-coding genes, 12S rRNA gene, 16S rRNA gene, and all tRNA genes, implemented in MrBayes (Ronquist et al. 2012) and RAxML (Stamatakis 2014). Outgroup taxa and close relatives of *Saurogobio* fishes were selected on the basis of results revealed in previous studies (Tang et al. 2011; Li et al. 2018). Our phylogeny showed that the genus *Saurogobio* was a monophyletic group and it could be divided into two major groups (Figure 1). One group included *S. dumerili*, *S. lissilabris*, *S. gymnocheilus*, and *S. immaculatus*, and another group consisted of *S. dabryi*, *S. gracilicaudatus*, and *S. xiangjiangensis.*

**Figure 1.**
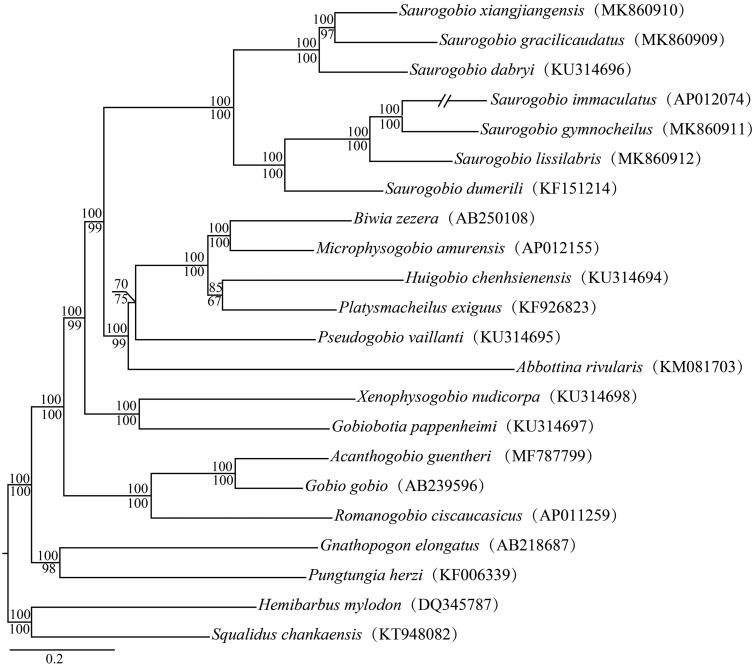
Phylogenetic relationships among *Saurogobio* fishes and their close relatives using 22 mitochondrial genomes. Shown are Bayesian posterior probabilities above branches for Bayesian analyses and bootstrap confidences below branches for maximum likelihood analyses. GenBank numbers are given in the parentheses.
